# Impaired Upper Airway Muscle Function with Excessive or Deficient Dietary Intake of Selenium in Rats

**DOI:** 10.3390/antiox13091080

**Published:** 2024-09-04

**Authors:** David P. Burns, Sarah E. Drummond, Stefanie Wölfel, Kevin H. Murphy, Joanna Szpunar, Ken D. O’Halloran, John J. Mackrill

**Affiliations:** 1 Department of Physiology, School of Medicine, College of Medicine and Health, University College Cork, T12 XF62 Cork, Ireland; d.burns@ucc.ie (D.P.B.); sarah.drummond@ucc.ie (S.E.D.); woelfel-stefanie@gmx.de (S.W.); kevinmurphy12@umail.ucc.ie (K.H.M.); k.ohalloran@ucc.ie (K.D.O.); 2 IPREM UMR 5254, CNRS, E2S UPPA, Université de Pau et des Pays de l’Adour, Hélioparc, 64053 Pau, France; joanna.szpunar@univ-pau.fr

**Keywords:** obstructive sleep apnoea (OSA), dietary selenium, selenoprotein N (SELENON), selenoprotein S (SELENOS), selenoprotein W (SELENOW), chronic intermittent hypoxia (CIH)

## Abstract

Obstructive sleep apnoea (OSA) involves impaired upper airway muscle function and is linked to several pathologies including systemic hypertension, daytime somnolence and cognitive decline. Selenium is an essential micronutrient that exerts many of its effects through selenoproteins. Evidence indicates that either deficient or excessive dietary selenium intake can result in impaired muscle function, termed nutritional myopathy. To investigate the effects of selenium on an upper airway muscle, the sternohyoid, rats were fed on diets containing deficient, normal (0.5 ppm sodium selenite) or excessive (5 ppm selenite) selenium for a period of two weeks. Sternohyoid contractile function was assessed ex vivo. Serum selenium levels and activity of the glutathione antioxidant system were determined by biochemical assays. The abundance of three key muscle selenoproteins (selenoproteins -N, -S and -W (SELENON, SELENOS and SELENOW)) in sternohyoid muscle were quantified by immunoblotting. Levels of these selenoproteins were also compared between rats exposed to chronic intermittent hypoxia, a model of OSA, and sham treated animals. Although having no detectable effect on selected organ masses and whole-body weight, either selenium-deficient or -excessive diets severely impaired sternohyoid contractile function. These changes did not involve altered fibre size distribution. These dietary interventions resulted in corresponding changes in serum selenium concentrations but did not alter the activity of glutathione-dependent antioxidant systems in sternohyoid muscle. Excess dietary selenium increased the abundance of SELENOW protein in sternohyoid muscles but had no effect on SELENON or SELENOS. In contrast, chronic intermittent hypoxia increased SELENON, decreased SELENOW and had no significant effect on SELENOS in sternohyoid muscle. These findings indicate that two-week exposure to selenium-deficient or -excessive diets drastically impaired upper airway muscle function. In the sternohyoid, SELENON, SELENOS and SELENOW proteins show distinct alterations in level following exposure to different dietary selenium intakes, or to chronic intermittent hypoxia. Understanding how alterations in Se and selenoproteins impact sternohyoid muscle function has the potential to be translated into new therapies for prevention or treatment of OSA.

## 1. Introduction

In obstructive sleep apnoea (OSA), the impaired function of upper airway dilator muscles results in intermittent full or partial collapse of the pharynx, oxygen desaturation and interrupted sleep [1]. This can lead to daytime somnolence, systemic hypertension and cognitive decline [2]. The underlying causes of upper airway muscle dysfunction in OSA have not been fully resolved, but ageing, obesity, diet, alcohol consumption, smoking, gender and genetics are all contributing factors [3].

Selenium is an essential dietary micronutrient. Dietary selenium has a narrow beneficial window, with a recommended daily allowance of 70 μg/day for men and 60 μg/day for women, with an upper tolerable limit of 400 μg/day [4]. Selenium exerts most of its physiological effects via the 21st amino acid selenocysteine, which is incorporated into a small set of proteins (25 in humans) via recoding of a UGA stop codon [5]. This involves multiple translational steps, initiated by a selenocysteine insertion sequence of mRNAs encoding selenoproteins, downstream of the UGA codon [6]. Endemic selenium deficiency occurs in geographical regions where the levels of the element in the soil are low, such as the Keshan district of China. In humans, dietary deficiency of selenium is associated with a number of disorders including increased mortality, type 2 diabetes, reduced immune response, increased risk of prostate and colorectal cancer, cognitive decline and Keshan disease, an endemic cardiomyopathy linked to coxsackie virus infection [7]. Dietary deficiency of selenium also results in a number of disorders of livestock, including white muscle disease, the necrosis and deposition of calcium salts in skeletal muscle [8]. Conversely, excessive selenium intake can also lead to adverse health effects, including alopecia, dermatitis, type 2 diabetes, muscle weakness, respiratory failure, death and increased risk of non-melanoma skin cancer and prostate cancer [7,9,10].

The protective effect of maternal Se intake against prenatal myopathy was first reported in ewes, in 1958 [11]. In Sprague-Dawley rats, 12 week feeding with a selenium-deficient diet resulted in decreased diaphragm glutathione peroxidase (GPX) activity [12]. In combination with the oxidative stress of inspiratory resistive loading, selenium-deficient diets also lead to impaired twitch and tetanic force production, and a downward shift in the force-frequency relationship in rat diaphragm [13]. In humans, dietary selenium deficiency is characteristic of elderly populations and is associated with negative outcomes [14]. In women over 65 years, low serum selenium concentrations are associated with decreased hand-grip strength [15]. In a longitudinal study of men over 85 years of age, low dietary selenium intake was linked with poor performance in hand-grip strength and timed up-and-go tests [16].

Selenoproteins of known function, such as GPXs and thioredoxin reductases, participate in redox reactions. Selenoprotein N (SELENON, previously named SEPN1) is present in vertebrate skeletal muscles, particularly during foetal development and is a transmembrane protein located in the endoplasmic reticulum (ER) and sarcoplasmic reticulum (SR) [17]. In humans, loss-of-function mutations in the *SELENON* gene result in a disorder called rigid-spine muscular dystrophy, characterized by axial muscle weakness, scoliosis, spinal rigidity and respiratory failure [18,19]. In contrast, mice lacking a functional *SELENON* gene do not display any major phenotype, except for reduced mobility and body rigidity following forced swimming tests [20]. SELENON influences excitation–contraction coupling in skeletal muscle via at least two mechanisms: (i) by acting as a redox sensor to regulate the gating of type 1 ryanodine receptor (RyR1) Ca^2+^-release channels in the SR [21]; and (ii) by controlling the activity of the SR/ER Ca^2+^-ATPase (SERCA) pumps through reduction of critical cysteine residues, thereby promoting Ca^2+^-loading of the SR [22]. Mice whose diets were supplemented with either selenate or selenium nanoparticles (0.5 ppm or 5 ppm Se, versus 0.3 ppm in control animals) displayed enhanced voluntary running, increased soleus (SOL) and extensor digitorum longus (EDL) muscle single-twitch amplitude, and augmented depolarization-induced Ca^2+^-release in flexor digitorum brevis muscle fibres [23]. Similar dietary supplementation of selenium improved voluntary exercise, ex vivo muscle performance, depolarization-induced Ca^2+^-release and increased SELENON protein levels in muscles from sarcopenic (aged) mice [24].

Two other ER-/SR-resident selenoproteins that regulate skeletal muscle contractile function are selenoprotein W (SELENOW) and selenoprotein S (SELENOS). SELENOW is an oxidoreductase whose protein abundance is increased by high dietary selenium, in both rats and pigs [25,26]. In the rat L8 skeletal myoblast cell-line, Se supplementation enhances levels of SELENOW protein by stabilizing its mRNA [26]. SELENOW promotes skeletal muscle differentiation by inhibiting the interaction between TAZ (transcriptional co-activator with PDZ-binding motif) and 14-3-3 protein [27]. In skeletal myoblasts from chickens treated with a selenium-deficient diet for 25–30 days, there was an enhanced Ca^2+^ leak from the SR and altered levels of several Ca^2+^-transporters and -channels. SELENOW knockdown using siRNA mimicked these effects of dietary selenium deficiency [28].

SELENOS is also known as VCP-interacting membrane protein (VIMP) or Tanis, is abundant in fast-twitch muscle and participates in ER-dependent protein degradation (ERAD) [29], a quality control system for the removal of misfolded proteins. In addition to ERAD, SELENOS also regulates the transport and maintenance of other multiprotein complexes, including the nuclear pore complex and the anaphase-promoting complex [30]. SELENOS^−/−^ or ^+/−^ knockout mice display reduced spontaneous physical activity and decreased force production by EDL muscles [31]. These effects were independent of inflammation or cellular stress, and potentially involved alterations in skeletal muscle microvasculature [32]. In contrast, genetic reduction of SELENOS (heterozygous knockouts) exacerbated the profile of inflammatory cytokines of fast-twitch muscle fibres from mdx mice, a model of Duchenne muscular dystrophy [33].

In humans, there are associations between selenium status and OSA. An anecdotal case report described the apparent cessation of sleep apnoeic events when a subject commenced selenium supplementation, as a treatment for arthritis [34]. A Taiwanese study of 44 patients with mild to moderate OSA and 20 control subjects revealed a negative correlation between erythrocyte selenium concentrations and the apnoea/hypopnoea index (AHI) [35]. In contrast, a Turkish case-control study revealed that following adjustments for age, gender and body mass index, plasma selenium concentrations were positively correlated with AHI [36]. The authors suggested that elevated plasma selenium might represent an adaptive antioxidant response in OSA.

These observations indicate that both dietary selenium intake and selenoprotein abundance influence skeletal muscle performance. There appear to be biphasic relationships between dietary selenium and measures of OSA. Gaps in current knowledge of this area include understanding the effects of dietary selenium intake on upper airway muscle function and the role that this, and selenoprotein levels, might play in OSA. The work presented here aims to examine the effects of dietary selenium deficiency or excess on rat sternohyoid muscle contractile performance and on the levels of three major selenoproteins in muscle (SELENON, SELENOS and SELENOW). It also seeks to investigate changes in the levels of these selenoproteins in the rat sternohyoid in response to chronic intermittent hypoxia, an animal model of OSA.

## 2. Materials and Methods

### 2.1. Materials

Analytical reagent grade chemicals were purchased from Sigma-Aldrich (Saint-Quentin Fallavier, France) or Merck Life Science (Carrigtwohill, County Cork, Ireland). Hydrogen peroxide from Fisher Scientific (Hampton, NH, USA) and nitric acid (INSTRA-ANALYZED) from Baker (Central Valley, PA, USA) were used for sample digestion. Water (18 MΩ cm) was obtained with a Milli-Q system (Millipore, Bedford, MA, USA). Selenium-deficient rat chow, or identical chow supplemented with either 0.5 ppm or 5 ppm sodium selenite, was supplied by Envigo (Belton, Leicestershire, UK).

### 2.2. Ethical Approval

Procedures on live animals were performed under license from the Government of Ireland Department of Health (B100/4498) in accordance with National and European legislation (2010/63/EU) following ethical approval by University College Cork (AEEC no. 2013/035).

### 2.3. Experimental Animals, Dietary Interventions and Chronic Intermittent Hypoxia

Adult male Wistar rats were purchased from Envigo UK and transferred to University College Cork’s animal facility. Following acclimation to the unit (>1 week), rats were fed for two weeks with selenium-deficient rat chow (n = 10), or identical chow supplemented with either 0.5 ppm (n = 10) or 5 ppm (n = 10) sodium selenite, supplied by Envigo (Belton, Leicestershire, UK). A dietary intervention of two weeks was selected, based on previous studies indicating overt changes to rodent skeletal muscle biochemistry or physiology in response to modified selenium intake within this time period. For example, GPX activity (GPX being a family of selenoproteins) in rat soleus muscle was significantly reduced within two weeks upon exposure to a selenium-deficient diet [37]. Conversely, beneficial effects of increased selenium intake on aged mouse skeletal muscle function were significantly improved within two weeks [24]. These changes occurred without detectable changes in whole body physiology, such as body weight. Finally, effects of other dietary interventions on muscle physiology, such as the effect of N-acetyl cysteine on mdx mouse respiratory muscles, have been reported to occur within 14 days [38]. During this experimental protocol, drinking water was available ad libitum.

Sternohyoid and diaphragm muscle samples were also obtained from adult male Wistar rats exposed to chronic intermittent hypoxia as part of a separate study. For daily gas treatment, rats were housed in standard cages placed in commercially designed hypoxia chambers (Oxycyler^TM^, Biospherix, Lacona, NY, USA). Exposure to chronic intermittent hypoxia consisted of the cycling of gas in environmental chambers from normoxia (21% O_2_) for 210 s to hypoxia (5% O_2_ at the nadir) over 90 s (12 cycles/h) for 8 h/day during light hours for 14 consecutive days. The sham group was exposed to 21% O_2_ in parallel.

### 2.4. Ex Vivo Sternohyoid Muscle Function

Rats were anaesthetised by exposure to 5% isoflurane in air and killed by cervical dislocation. Sternohyoid muscle was immediately excised and placed in a tissue bath at room temperature containing continuously gassed hyperoxic (95% O_2_/5% CO_2_) Krebs solution (in mM: NaCl, 120; KCl, 5; Ca^2+^ gluconate, 2.5; MgSO_4_, 1.2; NaH_2_PO_4_, 1.2; NaHCO_3_, 25; and glucose, 11.5) and d-tubocurarine (25 μM) prior to functional analysis. Muscle preparations were suspended vertically between two platinum plate electrodes in a water-jacketed tissue bath at 35 °C containing Krebs solution and were continuously gassed with hyperoxia (95% O_2_ and 5% CO_2_). One end of the muscle was anchored to a fixed hook and the other end was attached to a lever connected to a dual-mode force transducer (Aurora Scientific Inc.; Aurora, ON, Canada). To determine muscle optimum length (L_o_), the lengths of the muscle preparations were adjusted using a micro-positioner between intermittent twitch contractions. The muscle length which revealed the maximal isometric twitch force for a single isometric twitch stimulation (supramaximal stimulation, 1 ms duration) was considered L_o_. Sternohyoid muscle preparations were maintained at L_o_ for the duration of the protocol.

Experimental protocol: First, a single isometric twitch contraction was measured and peak isometric twitch force (P_t_), contraction time (CT) and half-relaxation time (½RT) were assessed. To examine the force–frequency relationship, muscle bundles were stimulated sequentially at 10, 20, 40, 60, 80, 100, 120, 140 and 160 Hz (300 ms train duration). Contractions were interspersed by a 1 min interval. Following the isometric protocol, an isotonic contraction was elicited in preparations at 0% load to examine maximum unloaded muscle shortening and velocity of shortening.

Data analysis: Muscle bundle cross-sectional area (CSA) was calculated by dividing muscle mass (weight in grams) by the product of muscle L_o_ (cm) and muscle density (assumed to be 1.06 g/cm^3^). Muscle force was divided by bundle CSA and expressed as specific force (N/cm^2^). CT and ½RT were measured as indices of isometric twitch kinetics and expressed in ms. For the measurement of muscle shortening velocity (V_max_), the distance shortened during the initial 30 ms of an unloaded contraction was assessed and determined in absolute units (cm/s) and was normalized to L_o_ and expressed in L_o_/s.

### 2.5. Muscle Histology

#### 2.5.1. Tissue Preparation

Sternohyoid samples were embedded in optimum cutting temperature embedding medium (OCT; VWR International, Dublin, Ireland) for cryoprotection and then frozen in isopentane (Sigma-Aldrich, Wicklow, Ireland) cooled on dry ice. Samples were then stored at −80 °C for subsequent structural analysis. Serial transverse muscle sections (10 µm) were cut using a cryostat (Leica CM3050; Leica Microsystems, Nussloch, Germany) at −22 °C and mounted across polylysine-coated glass slides (VWR International, Dublin, Ireland) allowing for a distribution of tissue on a given slide.

#### 2.5.2. Laminin Immunofluorescence

Laminin immunofluorescence was used to examine the fibre size (calculated as minimum Feret’s diameter) distribution of the sternohyoid muscle. Slides were removed from the −80 °C freezer containing a minimum of four sections per slide, from two distinct regions of muscle tissue for each animal. A hydrophobic barrier was created around the individual tissue samples using a hydrophobic pen (ImmEdge^TM^ Vector Labs, Peterborough, UK). Briefly, tissue samples were immersed in phosphate-buffered saline (PBS) (0.01 M) containing 1% bovine serum albumin (BSA) for 15 min followed by 3 × 5 min PBS rinses. This was followed by a 30 min wash in PBS containing 5% goat serum (Sigma-Aldrich, Wicklow, Ireland). Following 3 × 5 min PBS washes, rabbit anti-laminin antibody (Sigma-Aldrich, 1:500) diluted in 1% BSA in PBS was applied to the slides and incubated at 4 °C overnight in a humidity chamber. The next day, slides were washed for 3 × 5 min in PBS before the application of the corresponding secondary antibody, fluorescein isothiocyanate (FITC)-conjugated goat anti-rabbit secondary antibody (1:250, Sigma-Aldrich). Slides were incubated for 1 h in the dark at room temperature. Finally, slides were washed for 3 × 5 min rinses in PBS.

Data analysis: Muscle sections were viewed at ×10 magnification and images were captured using an Olympus BX51 microscope and an Olympus DP71 camera. Cell Sens™ (Olympus) was used to digitally capture the images. Images were taken at random areas across each muscle section with multiple muscle sections analysed per animal. Individual images of laminin labelled fibres were captured and processed using ImageJ software version 1.54j. From each muscle section, the number of fibres per area along with the minimum Feret’s diameter of the individual muscle fibres was analysed using a specialized ImageJ macro. Frequency histograms were constructed to illustrate the distribution of fibre size in muscle sections across groups in addition to the mean minimum Feret’s diameter. Mean fibre area was calculated. Data generated from multiple images of sternohyoid muscle sections were averaged per animal before computing group means.

### 2.6. Biochemical Assays

Lysates from rat skeletal muscle and other tissues were prepared as described previously [39]. Protein concentrations were determined using Bradford assays, with bovine serum albumin as a standard. Assays of glutathione reductase, glutathione peroxidase, total glutathione and oxidized to reduced glutathione (GSSG:GSH) ratio were measured as described [38].

#### 2.6.1. Western Blotting

Lysates (100 μg protein/lane) from rat tissues were resolved on either 7.5% (SELENON) or 15% (SELENOS and SELENOW) reducing SDS-PAGE minigels. For detection of SELENON, proteins were transferred onto nitrocellulose membranes at 70 V for 1 h and then immunostained with a 1:1000 dilution of anti-SELENON rabbit antiserum pAb MH4, as described previously [21]. For detection of either SELENOS or SELENOW, proteins were transferred at 50 V for 30 min, then blots were stained with Ponceau S and were photographed. Blots were then blocked for 1 h using 1% fish skin gelatine (FSG) in Tris-buffered saline containing 0.1% Tween 20 (TBST). SELENOS was detected using a 1:1000 dilution of rabbit antibody HPA010025 (Merck Life Science Ltd.), and SELENOW with a 1:1000 dilution of rabbit polyclonal antibody 600401A29 (Rockland Immunochemicals, Pottstown, PA, USA) in 1% FSG/TBST. Following overnight incubation at 4 °C, blots were washed three times over 15 min with TBST, prior to a 1 h incubation with a 1:10,000 dilution of IRDye^®^ 800CW donkey anti-rabbit IgG (Li-Cor Biosciences Ltd., Lincoln, NE, USA) in 1% FSG/TBST. Blots were washed three times over 15 min with TBST and immunostaining detected using an Odyssey XF imaging system (Li-Cor Biosciences Ltd.) using standard settings. Exposure times or antibody dilutions were adjusted to achieve linearity of detection; for example, see Supplemental Figure S1.

#### 2.6.2. Determination of Total Selenium in Plasma

Plasma samples (250 mg) were weighed and digested with 1.25 mL of a mixture (4:1, *v*/*v*) of nitric acid and hydrogen peroxide in a 50 mL polypropylene tube (DigiTube, SPC Science, Baie-d’Urfé, QC, Canada) using a Digi-Prep system from SCP Science (QC, Canada), with the following temperature program: 0.5 h up to 65 °C and 4 h at 65 °C. After cooling to room temperature, the digest was diluted with 10 mL of deionized water to decrease the HNO_3_ concentration. The diluted sample was analysed by inductively coupled plasma mass spectrometry (ICP-MS); an Agilent 7500ce (Agilent, Tokyo, Japan) was used, optimized daily using a tuning solution (containing 1 ppb of Y, Tl, Li in 2% nitric acid). The following isotopes were monitored: 76Se, 77Se, 78Se, 80Se and 82Se. The observed isotopic pattern matched the theoretical one, indicating the absence of interference. A 5 point calibration curve was constructed and all values obtained were within the linear range of detection. The control sample was SERO201405 Seronorm Trace Elements in Serum (Level 1) from SERO (Norway) and the values obtained were within the certified range. The detection limit for Se in serum was 20 ppb, with a limit of quantification of 40 ppb. The content of Se in each sample was calculated as the mean of results obtained for the five selenium isotopes [40].

### 2.7. Statistical Analysis

Statistical comparisons were made across the three experimental dietary groups by one-way ANOVA with post hoc tests when appropriate. Two-way ANOVA was used to compare the effects of two independent variables on a third continuous variable. Student’s *t*-test was used to compare pairs of mean values. Further details are provided in the legends of each of the figures. In all cases, a threshold of *p* < 0.05 was taken as statistically significant.

## 3. Results

### 3.1. Feeding Rats with Selenium-Deficient or -Excessive Diets Results in Corresponding Changes in Serum Selenium Content

**Figure 1:**
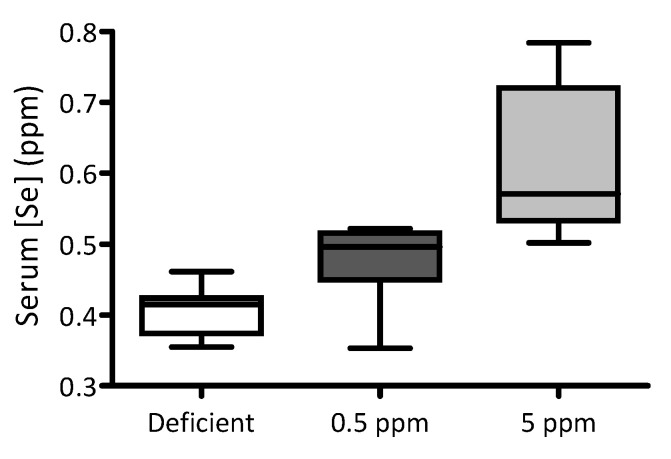
Impact of dietary Se intake on serum Se levels in rats. For two weeks, three groups of adult male Wistar rats were fed with either Se-deficient chow (nominally Se free), or that supplemented with either 0.5 ppm or 5 ppm sodium selenite (n = 10 for each group). The box-and-whisker plot shows the effect of these dietary interventions on serum selenium levels. Levels of Se were significantly different between groups (*p* < 0.0001 by one-way ANOVA; 0.5 ppm versus 5 ppm *p* < 0.05 and deficient versus 5 ppm *p* < 0.01, using Dunnett’s post hoc test).

Over a two-week period, adult male Wistar rats were fed with selenium-deficient chow, or identical chow supplemented with either 0.5 ppm (normal intake) or 5 ppm Se (excessive intake) in the form of sodium selenite. At the end of this period, the impact of dietary interventions on serum Se levels was quantified using ICP-MS. Serum Se concentrations correlate with the quantities of this element in the diet: the lowest levels were detected in the serum of animals fed with Se-deficient chow and the highest in those fed with chow supplemented with 5 ppm selenite, Figure 1.

### 3.2. Dietary Deficiency or Excess of Selenium Does Not Alter Body Mass or Organ Parameters

After two weeks of feeding with selenium-deficient, -standard (0.5 ppm Se) or -excessive (5 ppm Se) chow, the following characteristics of rats were measured: body mass, tibia length and the masses of the tibia, right ventricle, left ventricle, spleen, tibialis anterior, extensor digitorum longus, soleus, testes and thyroid. None of these parameters were statistically different between the three groups, Table 1.

### 3.3. Dietary Selenium Deficiency or Excess Both Influence Rat Sternohyoid Muscle Ex Vivo Performance

**Figure 2:**
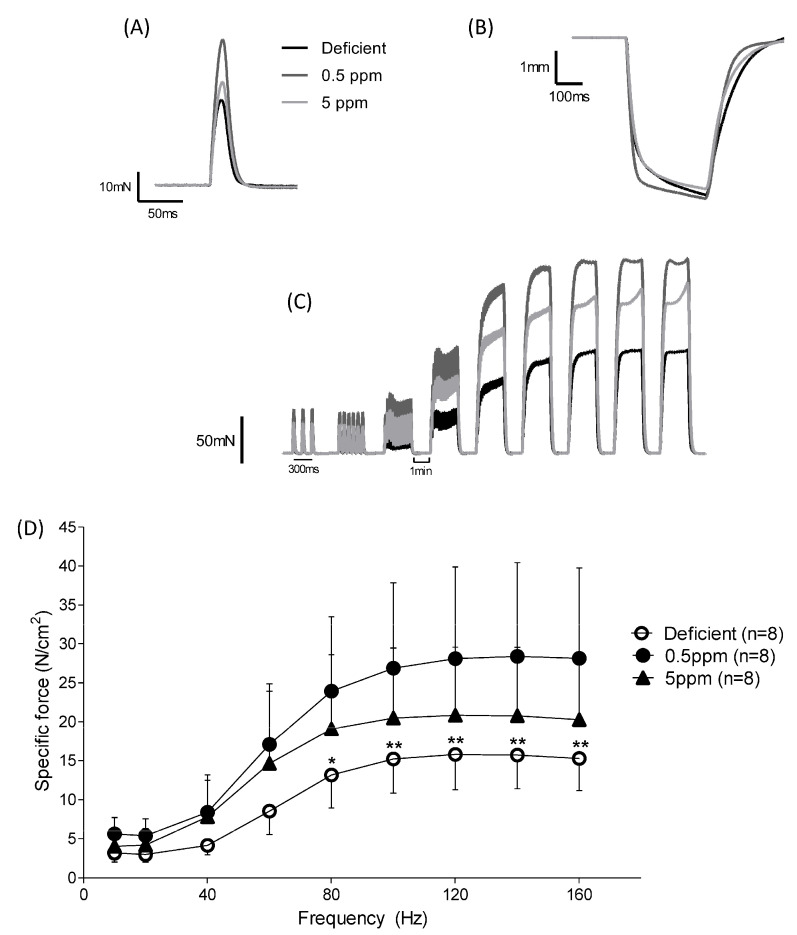
Dietary Se deficiency or excess has a detrimental impact on ex vivo contractile performance of rat sternohyoid muscle. The performance of SH muscle from rats fed with diets either deficient, adequate (0.5 ppm) or excessive (5 ppm) in selenium (n = 8 for each condition) was investigated ex vivo. For twitch kinetics, the time to peak (CT) was significantly reduced in deficient rats and significantly increased in 5 ppm Se diets, (panel **A**) and Table 2. The peak twitch tension (P_t_) was also significantly reduced in deficient rat SH relative to the 0.5 ppm controls, Table 2. Half-relaxation time was not significantly different between the three groups, (panel **B**). For specific force–stimulation frequency relationships, Se deficiency resulted in a significant reduction in peak isometric tetanic force relative to the control chow diet. Force–frequency relationships (original traces, panel **C**) and graphical presentation of mean data with SD (panel **D**) were compared using repeated measures two-way ANOVA; deficient versus 0.5 ppm, frequency: *p* < 0.0001, deficient: *p* < 0.0001, interaction: *p* < 0.0001 (80 Hz *p* < 0.05 (*), 100 Hz *p* < 0.01 (**), 120 Hz *p* < 0.01 (**), 140 Hz *p* < 0.01 (**), 160 Hz *p* < 0.01(**)); deficient versus 5 ppm, frequency: *p* < 0.0001, 5 ppm: *p* = 0.1262, interaction: *p* = 0.0801; 0.5 ppm versus 5 ppm, frequency: *p* < 0.0001, 5 ppm: *p* = 0.2491, interaction: *p* = 0.0322.

Although dietary deficiency or excess of selenium for two weeks had no significant effect on rat body parameters, it had significant effects on the ex vivo performance of the sternohyoid, a muscle involved in maintaining upper-airway patency. See Figure 2 and Table 2. These changes were a decrease (for deficient) or an increase (for 5 ppm Se) in time to peak contraction (CT), Figure 2A, and reductions in specific force generation at certain stimulation frequencies for SH muscle from both Se-deficient and 5 ppm rats (Figure 2C,D). However, half-relaxation time (½RT, Figure 2B) and peak shortening velocity (V_max_, Table 2) were unaltered by these dietary interventions.

**Figure 3:**
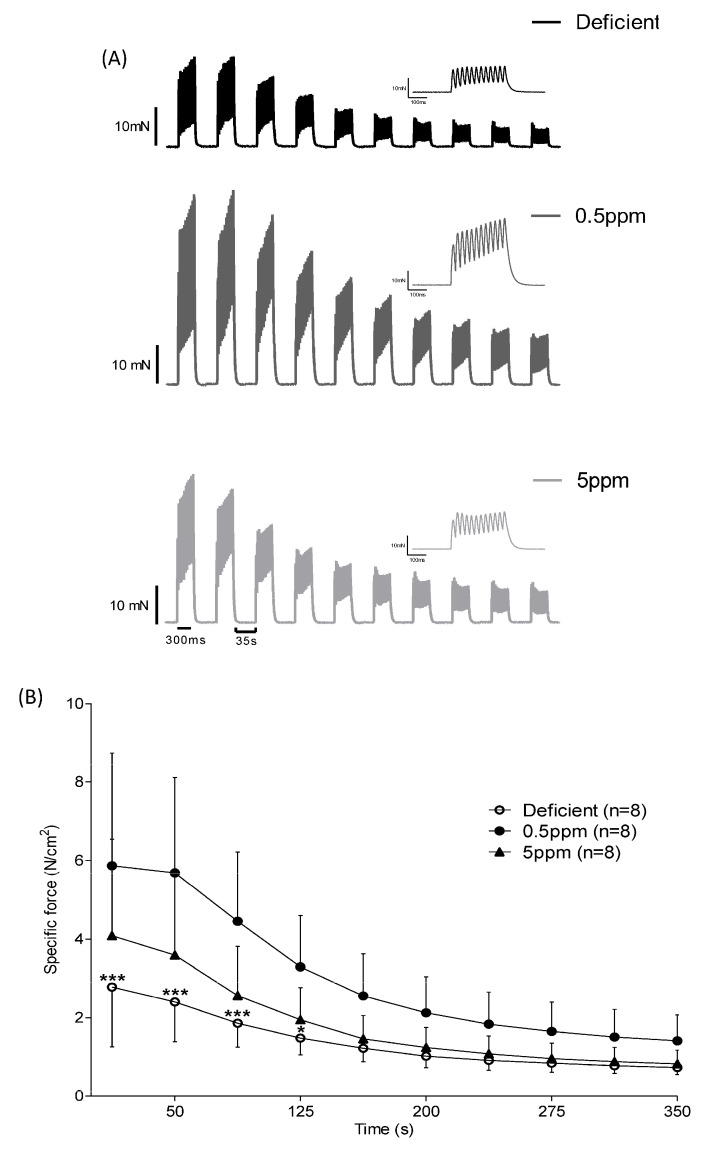
Effect of dietary Se deficiency or excess on rat sternohyoid contractile response to repeated stimulation. (Panel **A**) shows representative original traces of force production by SH muscle from deficient-, 0.5 ppm- or 5 ppm-Se fed rats, in response to repeat bouts of electrical stimulation. (Panel **B**) shows mean specific force generation versus time of stimulation (n = 8), including SD. By two-way ANOVA, time: *p* < 0.0001, deficient versus 0.5 ppm: *p* = 0.0042, 0.5 ppm versus 5 ppm: *p* = 0.0609, deficient versus 5 ppm: *p* = 0.2042; * *p* < 0.05, *** *p* < 0.001.

Measurement of isometric force production in response to repeated rounds of stimulation is used as an ex vivo simulation of fatigue. Over the first 125 s of repeated stimulation, SH muscle from rats fed with Se-deficient diets showed significantly reduced specific force generation relative to that from 0.5 ppm Se controls, Figure 3A,B.

### 3.4. Dietary Se Levels Do Not Alter SH Muscle Fibre Size

**Figure 4:**
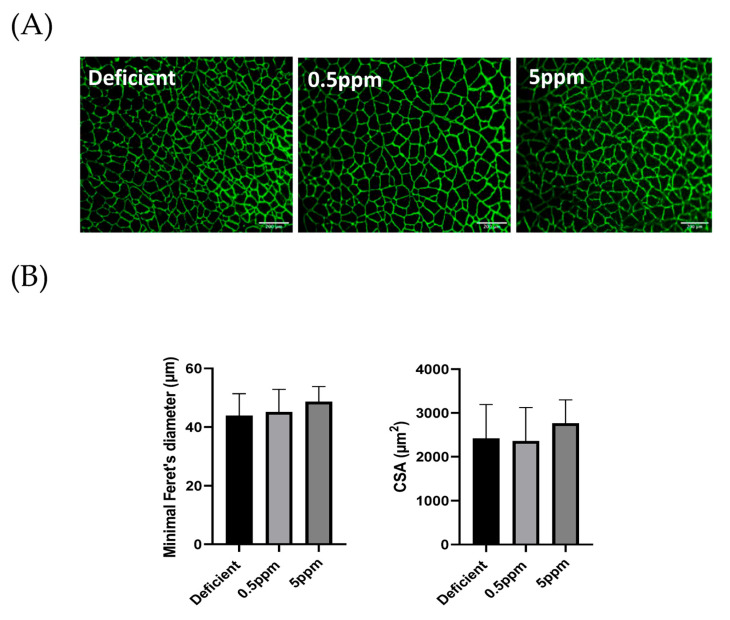
Se deficiency or excess does not affect rat sternohyoid muscle fibre size. Feeding rats with deficient, 0.5 ppm (adequate) or 5 ppm (excessive) Se diets for two weeks did not significantly alter SH muscle fibre size. (**A**) shows representative images of sternohyoid sections immunofluorescently labelled for laminin to show individual muscle fibres. (**B**) Data are shown as mean (SD) for Se-deficient (n = 7), control (0.5 ppm, n = 8) and Se-excess (5 ppm, n = 9). Data were statistically compared by one-way ANOVA for mean minimal Feret’s diameter (above) and mean cross-sectional area (CSA) Horizontal scale bars represent 200 μm.

Compared to 0.5 ppm controls, two weeks of deficient or excessive (5 ppm) Se diet did not significantly alter sternohyoid muscle fibre size determined by assessment of minimal Feret’s diameter. The mean minimal Feret’s diameter values and fibre cross-sectional area were similar between the three groups. Representative images are shown in Figure 4A and summary data are shown in Figure 4B.

### 3.5. Dietary Se Levels Do Not Alter Glutathione-Dependent Antioxidant Systems in Rat SH Muscle

**Figure 5:**
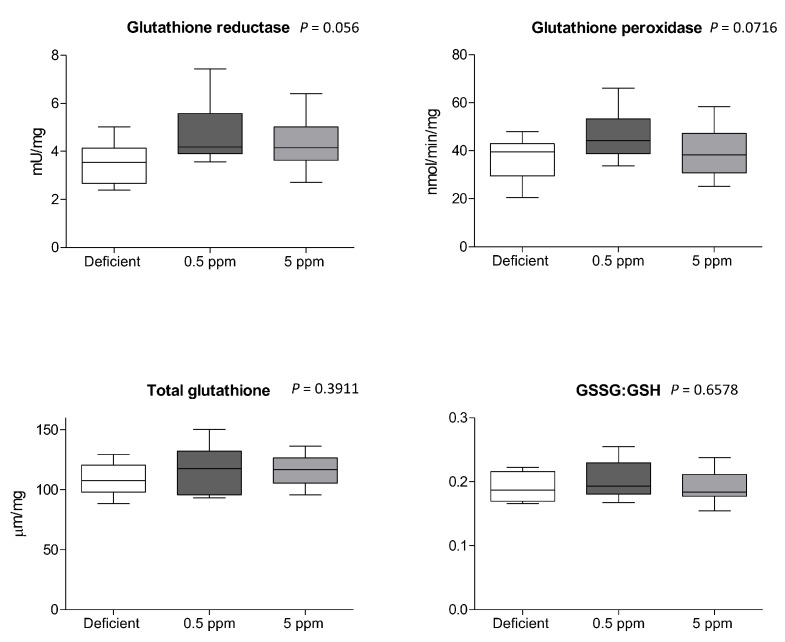
Effect of Se deficiency or excess on glutathione-dependent anti-oxidant systems in rat sternohyoid muscle. Feeding rats with deficient, 0.5 ppm (adequate) or 5 ppm (excessive) Se diets for two weeks did not significantly alter sternohyoid muscle glutathione reductase activity, glutathione peroxidase activity, total glutathione (all per mg protein), or the ratio between oxidized and reduced glutathione (GSSG:GSH). The box-and-whisker plots display mean values (n = 8) and SD. *p* values were determined by one-way ANOVA.

Compared to 0.5 ppm controls, two weeks of deficient- or excessive (5 ppm) Se diet did not significantly alter the activity of glutathione reductase or glutathione peroxidase, the concentration of glutathione, or the ratio between oxidized and reduced glutathione (GSSG:GSH), Figure 5.

### 3.6. Abundance of SELENON and SELENOS Proteins in Rat SH Muscle Are Not Altered by Dietary Se, Whereas SELENOW Is Increased by Excessive Se Intake

**Figure 6:**
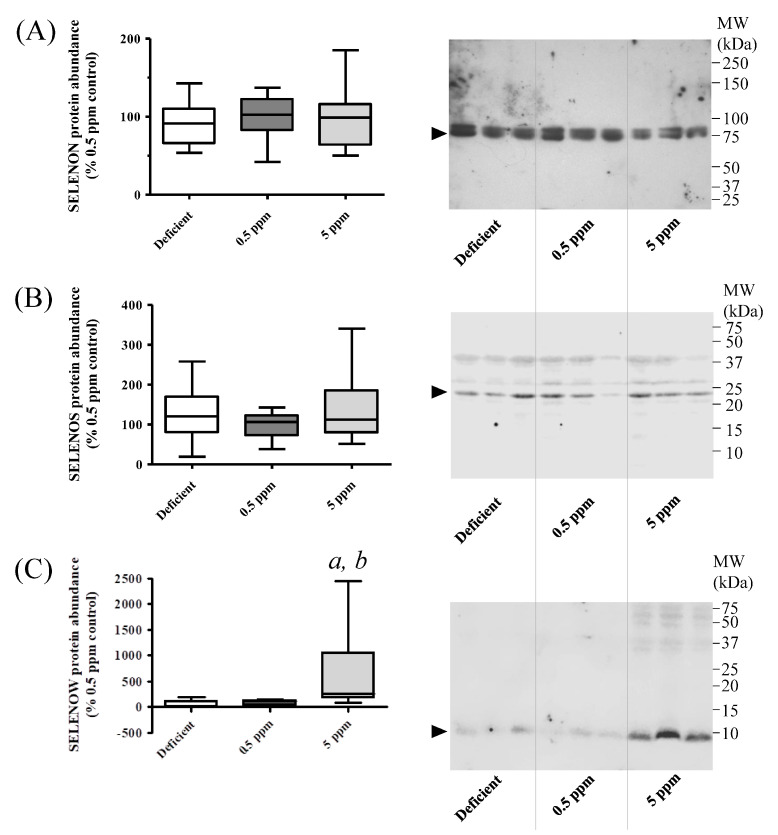
Effect of dietary selenium levels on rat sternohyoid muscle SELENON, SELENOS and SELENOW protein abundance. Sternohyoid muscle lysates (100 μg protein/lane) from the three dietary groups were resolved by 7.5% (**A**) or 15% (**B**) and (**C**) SDS-PAGE, transferred onto nitrocellulose and probed with rabbit antibodies against SELENON (**A**), SELENOS (**B**) or SELENOW (**C**). Immunodecorated proteins, indicated by arrows, were detected as described in Section 2.6.1. Protein densities were expressed as a ratio of the total protein loading (Ponceau S staining) and were normalized to the mean 0.5 ppm ratio. The graphs on the left-hand side of each panel show these normalized values (with SD, n = 10). One-way ANOVA indicated no significant differences between groups for SELENON and SELENOS, but in the 5 ppm Se group, SELENOW protein levels were significantly enhanced relative to Se-deficient (*a*, *p* < 0.001) or 0.5 ppm Se (*b*, *p* < 0.05) groups.

Detection of SELENON in SH muscle lysates in Western immunoblot assays revealed that deficient or excessive Se diets did not significantly alter the abundance of this protein, Figure 6A,B. In contrast, SELENOW protein levels were significantly increased in rats receiving excessive Se, relative to those consuming Se-deficient or normal diets, Figure 6C.

### 3.7. SH Muscle SELENON Is Increased and SELENOW Protein Is Decreased in a Rat Model of Obstructive Sleep Apnoea

**Figure 7:**
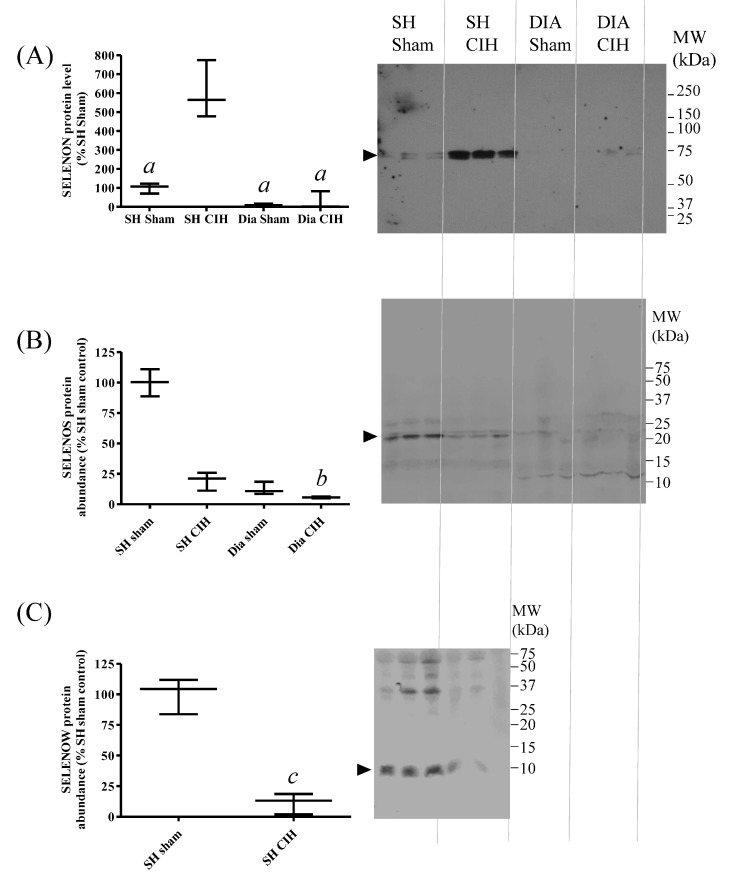
Effects of chronic intermittent hypoxia (CIH) on SELENON, SELENOS and SELENOW protein abundance in rat sternohyoid and diaphragm muscles. Western blots of sternohyoid (SH) and diaphragm (DIA) muscle lysates (100 μg protein/lane), from sham- or CIH-treated rats, were probed with antisera against SELENON (**A**), SELENOS (**B**) or SELENOW (**C**), as described in Figure 6. Immunoreactive band densities were divided by total protein densities (Ponceau S) and the mean values (n = 3) were graphed as mean values (with SD), normalized to the mean SH sham ratio. By one-way ANOVA and Tukey’s multiple comparison test, for SELENON, *a*, *p* < 0.001 versus SH sham; and for SELENOS, *b*, *p* < 0.01 versus SH sham. Note that SELENOW protein was not detectable in diaphragm; by Student’s *t*-test, *c*, *p* < 0.01 for SH sham versus SH CIH.

Chronic intermittent hypoxia (CIH) is used as a model of obstructive sleep apnoea (OSA). Given the potential association between Se status, nutritional myopathy and OSA, changes in SELENON, SELENOS and SELENOW protein abundance in response to CIH were investigated, in both SH and diaphragm (DIA) muscles. CIH led to a significant increase in SELENON (Figure 7A) and a decrease in SELENOW protein abundance (Figure 7C) in SH, compared with tissues from sham-treated animals. There were no significant changes in SELENOS protein levels between groups (Figure 7B), and SELENOW was not detectable in rat diaphragm.

## 4. Discussion

The central aim of this current study was to test the effect of deficient or excessive dietary selenium intake in rats, on the contractile performance of the sternohyoid, an upper airway dilator muscle. Effects of these dietary interventions on the abundance of key muscle selenoproteins (SELENON, SELEOS, SELENOW) were also investigated. In parallel, alterations in the levels of these selenoproteins were investigated in rats exposed to CIH, an animal model of OSA. The rationale underlying this is that associations between suboptimal dietary intake of selenium, reduced muscle performance and OSA have been reported in humans. Understanding the mechanistic links between selenium intake, selenoprotein abundance and contractile performance may unveil new therapeutic options for the management of OSA.

Previous epidemiological studies on distinct human populations have indicated either negative [35] or positive [36] correlations between serum Se concentration and AHI, a measure of OSA. One resolution of this discrepancy might be the narrow range between adequate Se intake (55–70 μg/day) and its upper tolerable limit (400 μg/day). Given that dietary intake is a major determinant of serum Se levels [5,7], the aim of this current study was to investigate if dietary Se deficiency (nominally Se-free) or excess (5 ppm sodium selenite) affected the physiology of rat SH, an upper airway dilator muscle. This current study also investigated changes in the levels of three selenoproteins (SELENON, SELENOS and SELENOW) in this muscle in response to dietary Se intake and in CIH, an animal model of OSA.

After two weeks of exposure to diets containing excess Se (5 ppm), rat serum Se was significantly elevated compared to rats consuming normal chow (0.5 ppm). Although the serum Se of rats consuming Se-deficient chow (nominally Se free) was lower than those consuming normal feed, this difference did not reach statistical significance. This is probably due to variability in serum Se levels between individual animals. Such variability could reflect differences in Se status between rats at the start of the study, or differences in their Se metabolism, including in the levels of the major storage and transport protein, SELENOP. Such differences in serum SELENOP concentrations between individuals have been reported in human populations [41]. Another confounding variable in this study is that rats were given free access to drinking water, which is an additional source of Se [42].

Within two weeks, dietary Se content exerted major effects on SH contractile properties. Se deficiency resulted in decreased time to peak twitch force, diminished peak twitch force, reduced tetanic force, depressed specific force generation over a range of stimulation frequencies and enhanced decline in force production in response to repeated stimulation causing fatigue. These changes occurred without any overt changes in body parameters, glutathione-dependent antioxidant systems or SH muscle fibre size. However, the severely depressed force-generating capacity of Se-deficient rat SH resembles those reported in mouse models of inherited myopathies, such as Duchenne muscular dystrophy in the *mdx* mouse [43]. In terms of SH muscle twitch kinetics, neither the peak contraction velocity nor the half-relaxation time were significantly altered in Se-deficient rats. This suggests that in rat SH muscle, neither the contractile apparatus nor mechanisms involved in relaxation (sarcoplasmic reticulum Ca^2+^-reuptake, troponin-I function) are dramatically altered as a result of dietary Se deficiency. Overall, this current work indicates that SH contractile function is an exquisitely sensitive biomarker of dietary Se deficiency, being drastically impaired within 2 weeks. This contrasts with the study of Matsuda et al., which reported that rats consuming Se-deficient diets for twenty weeks showed no significant reduction in muscle tone, as assessed by grip and rota-rod tests [37].

Excessive dietary Se also impacted SH physiology. In particular, the time to peak twitch force was significantly increased. The mechanisms by which excess dietary Se alter muscle function have not been fully delineated. For example, excessive dietary selenomethonine feeding of mice increases organ Se concentrations but did not alter the expression levels of the nine selenoproteins investigated. However, the non-selenoprotein protein-associated Se content of the liver and kidney was significantly increased [44]. This suggests increases in specific non-selenoprotein Se-binding proteins (the Se-binding proteins) [45] and/or increases in non-specific modification of proteins. Analyses of turkey and rat liver transcriptional changes in response to high Se diets failed to identify any shared transcripts associated with high Se status [46]. This supports the notion that, at least in liver, functional changes in response to high Se diets can occur through non-transcriptional processes.

Another mechanism by which deficient or excessive dietary Se intake might influence SH function is through modification of the function of selenoproteins that regulate Ca^2+^-release from the sarcoplasmic reticulum. Prominent among these is SELENON, which regulates Ca^2+^-release via ryanodine receptor channels [21,23] and Ca^2+^-reuptake by SERCA pumps [22]. SELENON protein might have key roles in SH muscle: the work presented here shows that it is particularly abundant in this tissue compared with other skeletal muscles. However, levels of SELENON protein were not detectably altered in SH muscle from Se-deficient or Se-excess rats. This contrasts with experiments on chickens, in which excessive dietary Se led to an increase in transcription of the SELENON gene [44]. However, our findings are consistent with a study on mice consuming an Se-deficient diet: although the total Se content of multiple organs was reduced, the levels of nine selenoproteins were not altered, with the exception of decreased GPX1, 2 or 3 in a restricted subset of tissues [44]. Furthermore, a 12-week intervention with deficient dietary Se in Sprague-Dawley rats also showed decreases in GPX activity and mRNA levels, in serum, liver and kidneys [12]. This aligns with the hypothesis of a selenoprotein hierarchy, in which the abundance of critical selenoproteins is preserved, even under conditions of severe dietary Se deficiency [5]. However, this current study conflicts with these observations, with no apparent change in serum GPX activity during 2 weeks of feeding with a Se-deficient diet. This suggests that nutritional myopathy might be an early consequence of Se deficiency [47].

CIH, an animal model of OSA, resulted in a significant increase in SELENON protein levels in SH muscle. Increased abundance of SELENON might represent an adaptive response to oxidative stress. In mice exposed to oxidative stress in the form of 8 months of exposure to a vitamin E-deficient diet followed by 5 days of 50 min running sessions, staining of tibialis anterior muscles for NADPH revealed significantly greater numbers of histological cores in SELENON^−/−^ animals relative to their wildtype counterparts [48]. This suggests that SELENON might be protective against muscle damage caused by oxidative stress.

In this current study, SELENOS protein abundance was not significantly altered by dietary Se deficiency, Se excess or by CIH. In contrast, SELENOW protein was increased by dietary Se excess and was reduced by CIH. This effect of dietary Se excess on SELENON is consistent with those reported previously in rat [49], pig [25] and chicken [50] skeletal muscle. In chickens, dietary Se supplementation of hens increased both muscle performance and SELENOW abundance in their offspring [51]. Given the decreased abundance of SELENOW in SH muscles exposed to CIH, dietary selenium supplementation might represent one means of reversing upper airway muscle weakness in OSA in humans. However, such an intervention would need to be carefully titrated, given the biphasic effects of dietary Se on SH muscle performance.

Another limitation of translating the work presented here to clinical applications are the physiological differences between humans and model animals. For example, loss-of-function mutations of SELENON can result in severe myopathies in humans. In contrast, the phenotype of SELENON-knockout transgenic mice is overtly detectable following muscle stress, such as during intense, prolonged exercise (forced swim-test) [20]. However, nutritional myopathy due to dietary Se deficiency is observed in many vertebrate species [10], suggesting that findings in rats might be translatable to clinical applications, such as prevention or treatment of OSA.

## 5. Conclusions

Overall, this current study indicates that either dietary Se deficiency or excess negatively impacts rat SH contractile function. This highlights the difficulties in titrating dietary Se as a therapeutic intervention in diseases such as OSA. The drastic changes in rat sternohyoid contractile performance following either deficient or excessive dietary selenium intake for two weeks did not involve overt changes in other organ systems. This suggests that sternohyoid physiology is exquisitely sensitive to dietary selenium, and that this might underlie pathological states, such as OSA. Furthermore, decreases in the contractile properties of the sternohyoid muscle did not involve alterations in fibre size distribution, nor changes in the level of SELENON or SELENOS proteins, but excess dietary Se was associated with increased SELENOW in this tissue. In sternohyoid muscle from a model of OSA, levels of SELENON protein were increased, SELENOW protein was decreased and there was no detectable change in SELENOS protein relative to sham controls. Uncovering the mechanisms by which dietary Se influences SH muscle contractile function could represent an important step in developing more specific therapeutic regimens targeting disorders of this representative upper airway muscle implicated in the control of airway patency.

## Figures and Tables

**Table 1:** Effect of feeding rats selenium-deficient, -standard (0.5 ppm) or -excessive (5 ppm) chow on body and tissue masses, and on tibia length. Data shown are mean values ± standard deviation (SD). Data which were normally distributed were compared using one-way ANOVA; data which were not normally distributed (Body mass, Tibia Length, Sol and Thyroid) were compared using the Kruskal-Wallis non-parametric test. Statistical significance was taken as *p* < 0.05. Abbreviations: RV, right ventricle; LV, left ventricle; TA, tibialis anterior; EDL, extensor digitorum longus; Sol, soleus.

	Deficient (n = 10)	0.5 ppm (n = 10)	5 ppm (n = 10)	*p*-Value
Body mass (g)	377.9 ± 26.0	387.9 ± 35.9	380.9 ± 30.1	0.8472
Tibia length (cm)	4.0 ± 0.1	3.9 ± 0.1	4.0 ± 0.1	0.5324
Tibia mass (mg)	558.3 ± 50.2	552.1 ± 61.1	579.6 ± 33.9	0.4410
RV (mg)	166.5 ± 21.0	172.2 ± 18.5	162.0 ± 22.5	0.5573
LV (mg)	629.7 ± 76.1	670.3 ± 68.9	629.7 ± 52.1	0.3044
Spleen (mg)	696.7 ± 109.0	701.6 ± 72.0	673.9 ± 91.9	0.7834
TA (mg)	782.3 ± 113.0	770.6 ± 69.8	785.5 ± 51.0	0.9112
EDL (mg)	175.2 ± 18.5	168.4 ± 14.1	171.5 ± 20.3	0.7118
Sol (mg)	161.7 ± 15.6	162.8 ± 25.5	160.1 ± 8.11	0.9678
Testes (mg)	1769 ± 171.5	1864 ± 131.7	1814 ± 156.8	0.4192
Thyroid (mg)	16.6 ± 2.0	15.4 ± 3.7	18.5 ± 6.9	0.3768

**Table 2:** Effect of dietary Se deficiency or excess on ex vivo rat sternohyoid muscle contractile parameters. Data shown are mean values ± standard deviation (SD). Data which were normally distributed (P_t_, P_o_, V_max_) were compared using one-way ANOVA with Newman-Keuls multiple comparisons; data which were not normally distributed (CT, ½RT) were compared using the Kruskal-Wallis test with Dunn’s multiple comparisons. Statistical significance was taken as *p* < 0.05; ^a^ Deficient versus 0.5 ppm, *p* < 0.05; ^b^ Deficient versus 5 ppm, *p* < 0.05. Abbreviations: CT, time to peak contraction; ½RT, half-relaxation time; P_t_, peak twitch tension; P_o_, peak isometric tetanic force; V_max_, peak shortening velocity normalised to optimal length.

	Deficient (n = 8)	0.5 ppm (n = 8)	5 ppm (n = 8)	*p*-Value
CT (ms)	13.7 ± 0.8	14.8 ± 0.9	15.4 ± 1.5	0.0064 ^a,b^
½RT (ms)	8.4 ± 1.1	8.8 ± 1.2	9.5 ± 2.0	0.6921
P_t_ (N/cm^2^)	3.4 ± 1.2	5.9 ± 2.0	4.3 ± 1.8	0.0292 ^a^
P_o_ (N/cm^2^)	24.2 ± 7.6	39.8 ± 13.8	28.2 ± 11.3	0.0285 ^a^
V_max_ (L_o_/s)	8.0 ± 2.2	8.7 ± 0.9	8.7 ± 2.4	0.6929

## Data Availability

Data are contained within the manuscript.

## Supplementary Materials

The following supporting information can be downloaded at: https://www.mdpi.com/article/10.3390/antiox13091080/s1, Supplemental Figure S1: Linearity and specificity of Western immunoblot detection of SELENON in rat sternohyoid muscle.
