# Supplementary material for: Impaired Upper Airway Muscle Function with Excessive or Deficient Dietary Intake of Selenium in Rats
Source: Antioxidants (Basel). 2024 Sep 4;13(9):1080. doi: 10.3390/antiox13091080 (PMC11429047; doi:10.3390/antiox13091080)
Supplement: Supplementary file 1 [file antioxidants-13-01080-s001.zip › antioxidants-3152039-supplementary.pdf]

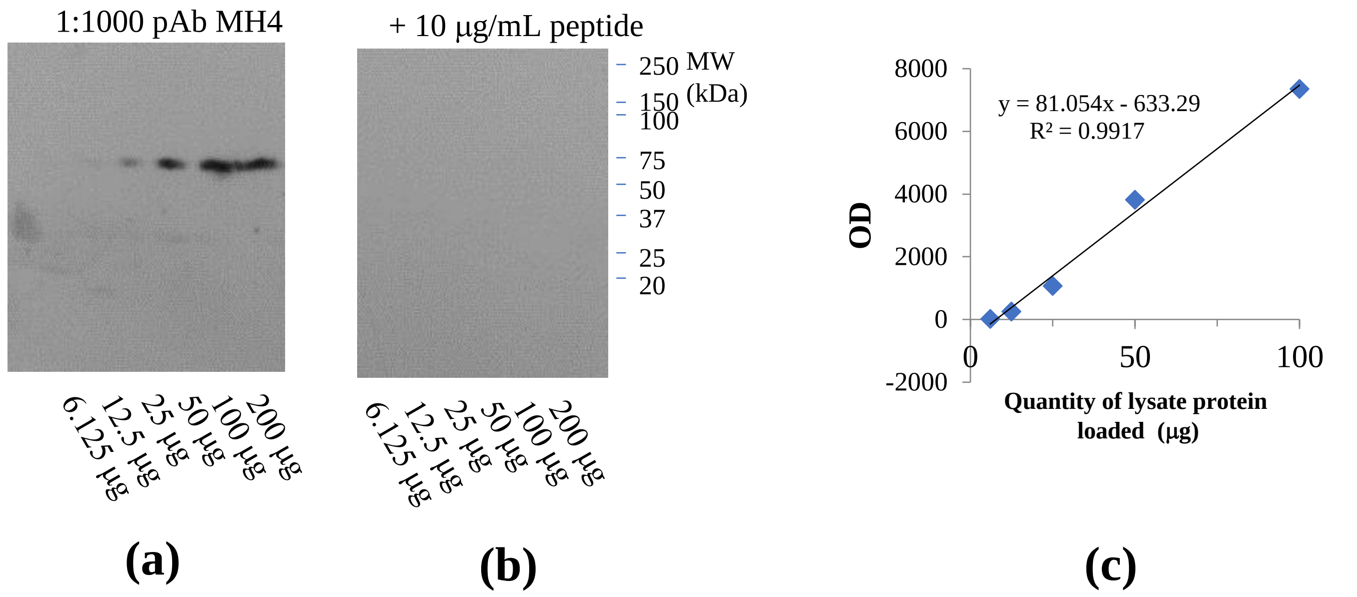

Supplemental Figure S1. Linearity and specificity of Western immunoblot detection of SELENON in rat sternohyoid muscle. In Western immunoblot assays, different amounts of sternohyoid lysate protein were probed with **(a)** a 1:1000 dilution of pAb MH4; or **(b)** the same antibody in the presence of 10 μg/mL of the peptide antigen used to generate the antiserum. Panel **(c)** was generated from the optical densities of the bands in panel **(a)** plotted against the amount of lysate protein loaded.
